# Supplementary material for: Comparative multiomics analysis of cell physiological state after culture in a basket bioreactor
Source: Sci Rep. 2022 Nov 23;12:20161. doi: 10.1038/s41598-022-24687-4 (PMC9686226; doi:10.1038/s41598-022-24687-4)

3666071.1:10603383:10603427:+@NW_023666071.1:10605875:10606165:+@NW_023666071.1:10606091:1060[illegible]

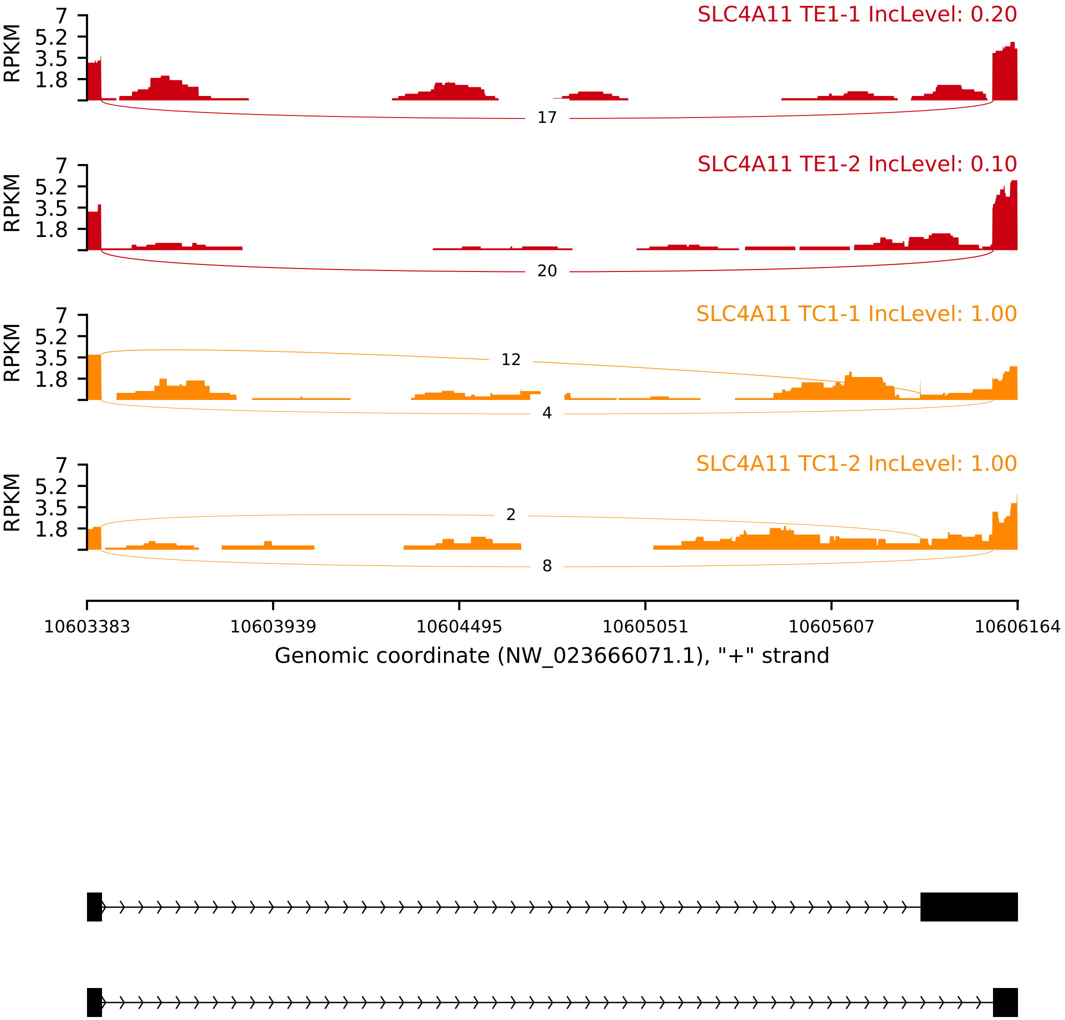

Supplement: Supplementary file 1 — Supplementary Information 1. [file 41598_2022_24687_MOESM1_ESM.zip › raw data/Transcriptomic raw data/7.AS/2.dasplot/TE1vsTC1/A3SS/2_SLC4A11_NW_023666071.1_10603383_10603427_+@NW_023666071.1_10605875_10606165_+@NW_023666071.1_10606091_10606165_+.pdf]
